# Bilateral Endogenous Bacterial Endophthalmitis Secondary to Methicillin-Resistant Staphylococcus aureus (MRSA) Bacteremia

**DOI:** 10.7759/cureus.29418

**Published:** 2022-09-21

**Authors:** Hannah Darland, Bryce Schutte, John Horne, Julie Jerabek, Paul G Millner

**Affiliations:** 1 Internal Medicine, Creighton University School of Medicine, Omaha, USA; 2 Infectious Diseases, Creighton University School of Medicine, Omaha, USA; 3 Internal Medicine/Infectious Diseases, Creighton University School of Medicine, Omaha, USA

**Keywords:** disseminated bacteremia, septic emboli, mrsa bacteremia, endogenous endophthalmitis, bilateral endophthalmitis

## Abstract

Endogenous bacterial endophthalmitis (EBE) is a rare but vision-threatening complication of bacteria spreading contiguously through the blood-ocular border. The condition is frequently associated with a poor visual prognosis and has even resulted in death. We present a case of a 48-year-old female with bilateral endogenous methicillin-resistant Staphylococcus aureus (MRSA)endophthalmitis further complicated by septic emboli, endocarditis, osteomyelitis, and septic polyarthralgia. Her vision did not return despite aggressive antibiotic therapy and urgent bilateral vitrectomy. This case presents an interesting and rare clinical vignette resulting in infection and damage of multiple organ systems that required aggressive management and carefully orchestrated multidisciplinary care.

## Introduction

Endogenous bacterial endophthalmitis (EBE) is a rare but severe ophthalmic emergency, which involves a vision-threatening complication of bacteria spreading contiguously through the blood-ocular border. It is often associated with a poor visual prognosis and has even resulted in death in its most severe form [1-3]. Endogenous etiology accounts for only 2-8% of all cases of endophthalmitis [2], with approximately 12% of those cases being bilateral [4]. We report a case of methicillin-resistant Staphylococcus aureus (MRSA) bacteremia complicated by bilateral endogenous endophthalmitis and polyarthralgia in a 48-year-old diabetic female treated with intraocular and systemic antibiotics.

## Case presentation

**Figure 1:**
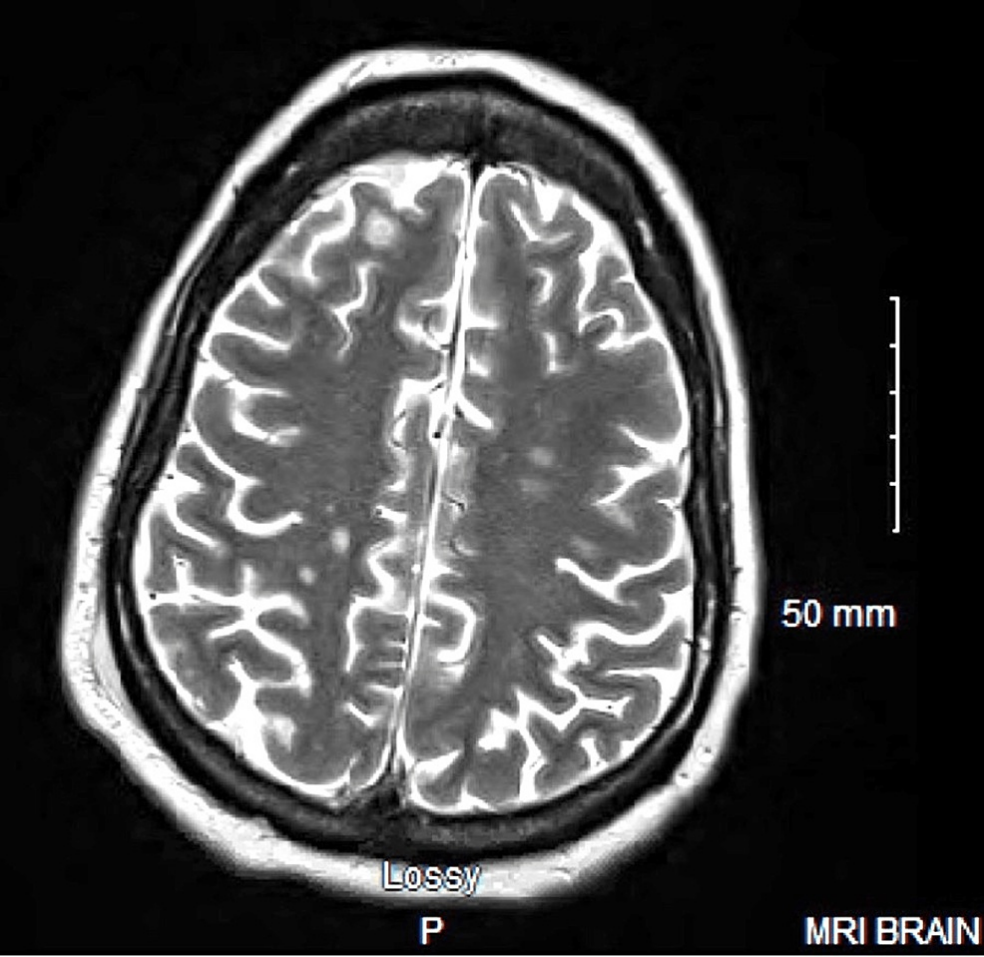
T2-weighted brain MRI The image demonstrates too-numerous-to-count areas of ring-enhancing lesions within the frontal, parietal, right occipital, brainstem, and cerebellar regions. Ring-enhancing mass seen within the right parietal scalp region concerning for abscess MRI: magnetic resonance imaging

A 48-year-old female with a past medical history of type II diabetes mellitus, diabetic retinopathy, hypertension, and peripheral vascular disease presented to a rural clinic for sudden vision loss with minimal light perception in both eyes. MRI of the brain was significant for small multifocal septic emboli (Figure 1), and a transesophageal echocardiogram (TEE) was negative for thrombus or vegetation. Initial lab values were significant for an HbA1c of 12.7, GFR of 23, CRP >375, and urinalysis was significant for microhematuria, glucosuria, granular casts, and 50-100 WBCs. Two blood cultures were positive for MRSA. The patient was started on IV vancomycin, linezolid, and ceftaroline for systemic MRSA coverage, and was shifted to our institution for further evaluation of her endophthalmitis.

Upon consultation, the ophthalmologist found visual acuity to be limited to hand motion 6 inches away bilaterally with significant conjunctival chemosis and normal intraocular pressure, and red reflex bilaterally. Due to the serious nature of her condition, the ophthalmologist injected intraocular vancomycin and ceftazidime after obtaining vitreous humor cultures at the bedside. These cultures were negative, but a high level of suspicion remained for endophthalmitis as the patient had received broad-spectrum antibiotics prior to culture with no alternative etiology apparent on evaluation. The patient underwent a bilateral pars plana vitrectomy the following day, which resulted in severe inflammation that was well controlled with prednisolone eye drops. Intraocular antibiotics were continued for the course of the hospital stay, and prognostic discussions about the unlikely return of vision occurred throughout her stay. Upon her discharge on hospital day 54, the patient did not have a return of vision in either eye. The final ophthalmology exam showed bare light perception of the left eye, no perception in the right eye, and dense vitreous debris with minimal red reflex, with future vision improvement appearing unlikely.

On admission and throughout the first several weeks, the patient endorsed severe pain in all four extremities at rest, as well as worsening pain with movement. The patient reported that she had been taking a considerable amount of non-steroidal anti-inflammatory drugs (NSAIDs) to help with her progressively worsening pain prior to hospital admission. Initial labs indicated acute kidney injury (creatinine: 2.4, BUN: 48), thereby limiting any contrast imaging. Renal function continued to deteriorate, which complicated her antibiotic course. Ultimately, the patient received ceftaroline and daptomycin for MRSA dual therapy.

**Figure 2:**
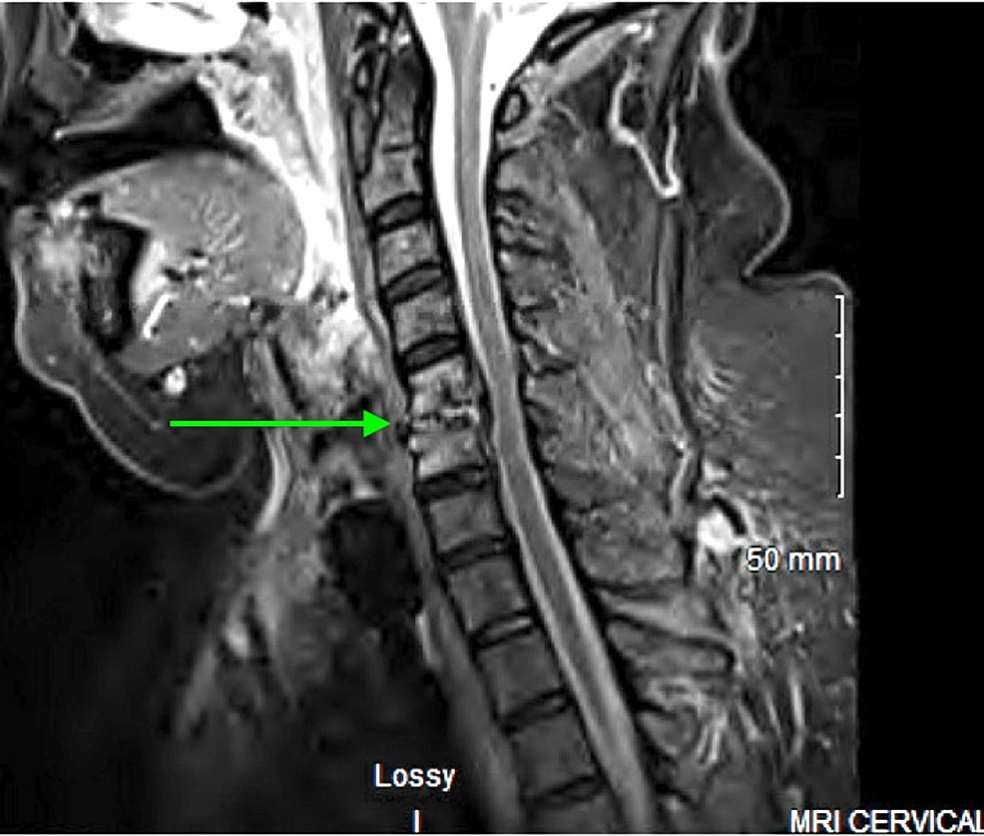
STIR-sequence cervical spine MRI demonstrating C5-C6 osteomyelitis STIR: short tau inversion recovery; MRI: magnetic resonance imaging

On hospital day six, blood cultures remained positive for MRSA, but renal function had improved with the cessation of NSAIDs and vancomycin (creatinine: 1.16, BUN: 37). A series of contrast scans and a repeat TEE were ordered to evaluate for possible sources of infection. TEE was positive for a 1-centimeter vegetation on the posteromedial papillary muscle of the mitral valve. CT imaging showed an intramuscular abscess in the left thigh as well as proximal and distal left triceps. Abscesses were drained with the resulting cultures growing MRSA. Imaging also revealed bilateral knee effusions. MRI of the cervical spine showed osteomyelitis at the level of C5-C6 (Figure 2).

**Figure 3:**
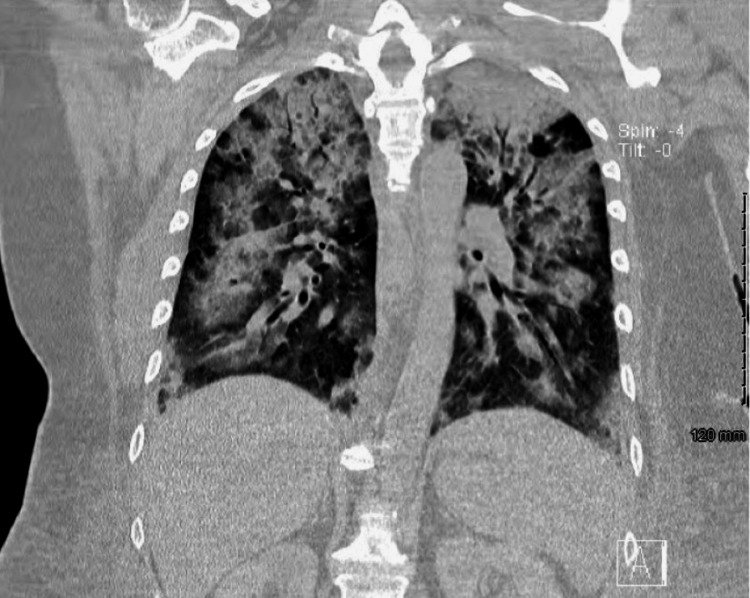
CT chest without contrast The image demonstrates bilateral pleural effusions, patchy mixed ground-glass and consolidative opacities, nonobstructive mixed atelectasis, and consolidation in the lung bases CT: computed tomography

Following sedation so that CT images could be obtained, oxygen requirements drastically increased from 3 liters per minute (LPM) by nasal cannula to a high-flow blender, with chest CT showing severe multifocal airspace disease (Figure 3). In the following days, pleural effusion was noted, and thoracentesis yielded 1.1 L of serosanguinous transudative fluid. Daptomycin was suspected as the cause of the deteriorating pulmonary status and was discontinued, leaving only ceftaroline for antibiotic therapy. A 12-day course of oral prednisone taper for daptomycin pneumonitis was also started.

After multiple positive blood cultures, her MRSA bacteremia cleared on hospital day 18, which was confirmed with negative repeat blood cultures on day 22 of hospitalization. Starting on the day of negative cultures, a six-week course of IV ceftaroline was chosen for the treatment of the MRSA bacteremia, endocarditis, osteomyelitis, and abscesses. Repeat TEE on hospital day 33 showed that the cardiac vegetation had significantly decreased in size, negating the need for surgical intervention with vegetation removal and papillary muscle replacement. Respiratory function gradually improved until the patient was back to room air.

As the infection cleared, the patient’s mobility improved rapidly. Based on her preference, arrangements were made to complete her antibiotic course and other follow-up care out of state.

In preparation for discharge on hospital day 54, a peripherally inserted central catheter (PICC) was placed for antibiotic administration. The patient was subsequently discharged to a skilled nursing facility in her home state.

## Discussion

Bilateral EBE is an exceedingly rare condition with significant long-term consequences including vision loss and possible mortality. This case presents bilateral EBE as a complication of MRSA bacteremia in a diabetic woman. In this patient, difficulty in achieving source control contributed to numerous sequelae including septic emboli, intramuscular abscess formation, septic polyarthralgias, and multiple organ dysfunction.

There are few cases of bilateral EBE reported in the literature, with even fewer reports of severe systemic infection. In the largest review of EBE cases to date (Jackson et al., 2014), patient characteristics were identified and reported. In this study, EBE had a high association (60% of cases) with underlying medical conditions including diabetes mellitus, IV drug use, underlying malignancies, and autoimmune conditions [1]. Additionally, 64% of patients were found to have an extraocular source of infection, with the most common sites identified as liver, lung, and endocardium [1]. In western countries, gram-positive infections predominated as the causative organisms [1].

A study by Steeples and Jones reported osteomyelitis in association with EBE [3]. A review of the literature did not identify other cases of bilateral EBE with other significant associations. According to Romero et al., vision is only preserved in 40% of endogenous endophthalmitis cases, even with aggressive IV antibiotic therapy [5]. Typical treatment of EBE is often based on the severity and location of other ongoing sources of infection, but in general, systemic therapy with a third-generation cephalosporin or fluoroquinolone with good intraocular penetration is recommended [6]. Pars plana vitrectomy is also a mainstay of treatment to reduce intraocular disease management and improve long-term outcomes [7].

Although the patient’s vision did not improve, aggressive antibiotic therapy with close monitoring for side effects was key to the stabilization and management of her multisystem infection. Poor blood sugar control with pre-existing diabetic neuropathy and an HbA1c of 12.7 may have contributed to the rapid vision loss in this case. A multidisciplinary approach was vital for the stabilization and management of this patient, requiring joint efforts from infectious disease, pulmonology, cardiology and cardiothoracic surgery, neurology, nephrology, internal medicine, social work, and numerous allied healthcare professionals.

## Conclusions

Physicians need to be aware of this rare but severe and vision-threatening complication of bacteremia. While it is most commonly noted in patients with gram-positive bacteremia and risk factors including type II diabetes mellitus, IV drug use, malignancy, and autoimmune conditions, any bacteremic patient with eye pain or vision changes should be evaluated promptly for EBE. Urgent ophthalmology consultation is warranted to obtain vitreous culture, administer ocular antibiotics, and rule out other pathologies.
